# Case Report: Triple gastro-colic fistula after percutaneous endoscopic gastrostomy placement

**DOI:** 10.3389/fsurg.2025.1681864

**Published:** 2025-11-19

**Authors:** Matteo Pittacolo, Marco Brolese, Arianna Vittori, Daniele Passeri, Renato Salvador, Valeria Valli, Lorenzo Vallese, Nicola Baldan, Gianfranco Da Dalt, Michele Valmasoni, Alberto Friziero

**Affiliations:** 1st General Surgery, Acute Care Surgery Unit, Department of Surgery, Oncology and Gastroenterology, University of Padova, Azienda Ospedale Università Padova, Padova, Italy

**Keywords:** gastrostomy, complications, intestinal perforation, fistula, large bowel resection

## Abstract

**Background:**

Percutaneous endoscopic gastrostomy (PEG) is a widely accepted procedure for long-term enteral nutrition. Although generally safe, rare but life-threatening complications can occur. We report a unique case of a triple gastro-colic fistula, identified during an emergency surgical intervention after radiological replacement of a PEG one year following its initial placement.

**Case presentation:**

An 83-year-old man with Parkinson's-related dysphagia underwent PEG placement. One year later, following catheter occlusion, it was replaced radiologically. The next day, the patient developed abdominal pain and diarrhea, and imaging revealed catheter misplacement into the transverse colon. Surgical exploration identified three chronic and dehiscent fistulous tracts involving the stomach, transverse colon, and sigmoid colon. The patient was treated with colonic resection, gastric double-layer suture, and surgical gastrostomy. Recovery was uneventful and the patient was discharged on postoperative day eight.

**Conclusion:**

This is the first reported case of a triple gastro-colic fistula following PEG placement. The case highlights that early recognition and multidisciplinary management of PEG-related complications are crucial. Prompt diagnosis and the availability of a specific Acute Care Department are essential for the effective management of such complex scenarios.

## Introduction

Long-term enteral nutrition in frail patients unable to maintain adequate oral intake is widely recognized as a safer alternative to parenteral nutrition, as it preserves bowel mucosal integrity and trophism, thereby reducing the risk of bacterial translocation and subsequent sepsis. While nasogastric tubes are generally preferred for short-term enteral nutrition due to their ease of placement and minimally invasive nature, gastrostomy becomes the method of choice when long-term enteral feeding is required (>4 weeks), as it offers a better-tolerated and effective solution (1, 2). Gastrostomy refers to the creation of an artificial external opening into the stomach, and the main techniques used to perform it include percutaneous endoscopic gastrostomy (PEG), radiologically inserted gastrostomy (RIG), and surgical gastrostomy (3). The choice of technique depends on the patient's anatomy, underlying condition, and institutional expertise. PEG was first described in 1980 for children with an inability to swallow and has since become the most widely adopted method, primarily due to its minimally invasive nature and favorable safety profile (4). According to the American Society for Gastrointestinal Endoscopy, PEG is the preferred approach in most patients requiring long-term enteral access, particularly when the indication is a medical or neurologic dysphagia rather than a surgical or anatomic barrier (3). Nonetheless, it is contraindicated mainly in patients with hemodynamic instability, severe coagulopathy, partial or subtotal gastrectomy and colonic interposition (5). PEG is performed endoscopically, with the feeding tube placed through the abdominal wall into the stomach, most commonly using the transoral “pull” technique. Alternative methods, such as the transoral “push” and transcutaneous “direct” (introducer) techniques, are also available, and are typically reserved for specific clinical scenarios (3). Differently, RIG is performed under imaging guidance (e.g., fluoroscopy) and it is generally reserved for patients in whom PEG is technically challenging or contraindicated, such as those with upper aerodigestive tract obstruction or aberrant anatomy. Surgical gastrostomy, now rarely performed, is typically limited to scenarios where percutaneous approaches are not technically viable (6–8). PEG has been consistently associated with a lower incidence of major adverse events, including colonic perforation, peritonitis, peristomal infections, significant bleeding necessitating transfusion, and in-hospital mortality. Evidence from large-scale databases and meta-analyses indicates that RIG is linked to a higher frequency of device-related issues, such as tube dislodgement or occlusion (smaller-diameter tubes and less secure fixation), while surgical gastrostomy is associated with the greatest risk of serious complications, including bowel perforation, infection, and death (3, 9). However, despite being generally safe and widely used, PEG carries inherent risks. Early complications primarily include tube dislodgement and occlusion, ileus, peristomal infection, bleeding, and peritonitis. Rare but serious complications encompass buried bumper syndrome, aspiration pneumonia, and colon perforation (10–12). Our study reports a case of a complex, multiple gastrocolic fistula diagnosed during the replacement of a PEG catheter, one year after its initial placement. A brief literature check using PubMed and Google Scholar, using “gastrostomy” and “fistula” as search terms, identified only single gastrocolic or gastrocolocutaneous fistulae after PEG, most often revealed during tube replacement. To our knowledge, no previous report has described a triple-fistula configuration. “Triple fistula” refers to three distinct organs involved in the fistula formation (i.e., stomach, transverse colon, and sigmoid colon) corresponding intra-operatively to five perforations.

## Case description

An 83-year-old man with a history of Parkinson's disease and prior hemorrhagic stroke underwent percutaneous endoscopic gastrostomy (PEG) for dysphagia in March 2023 at an outside center. Before PEG placement, the patient had progressive dysphagia with recurrent aspiration and weight loss. He also had chronic constipation consistent with Parkinson-related dysmotility. His therapy included levodopa and low-dose anticholinergics, both potentially reducing bowel motility and increasing the risk of visceral interposition during PEG procedures.

**Figure 1:**
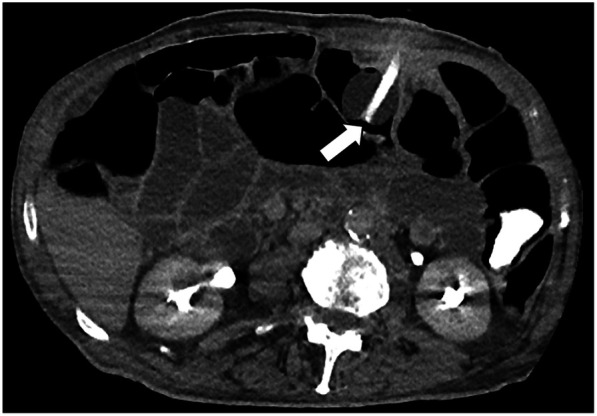
Computed tomography scan revealed the inflated balloon (white arrow) of the percutaneous endoscopic gastrostomy catheter located in the transverse colon after radiological replacement.

**Figure 2:**
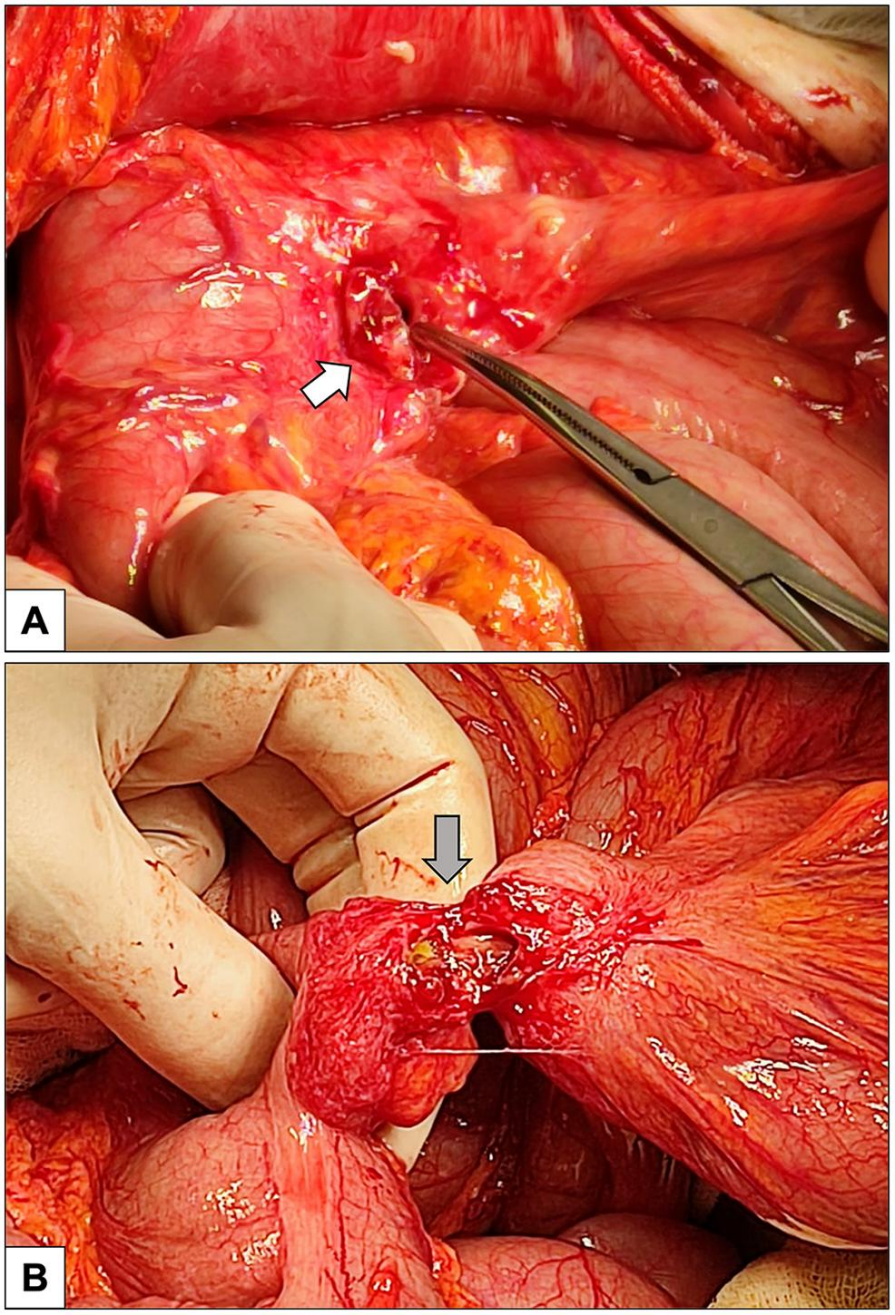
Intraoperative pictures. **(A)** Percutaneous endoscopic gastrostomy (PEG) entry site on the anterior wall of the stomach (white arrow). **(B)** Fistulous tract (gray arrow) created by PEG connecting the transverse colon (on the left) and the sigmoid colon (on the right). The visceral tissue adjacent to the dehiscent orifice exhibits pronounced inflammation, indicative of evolving chemical peritonitis.

**Figure 3:**
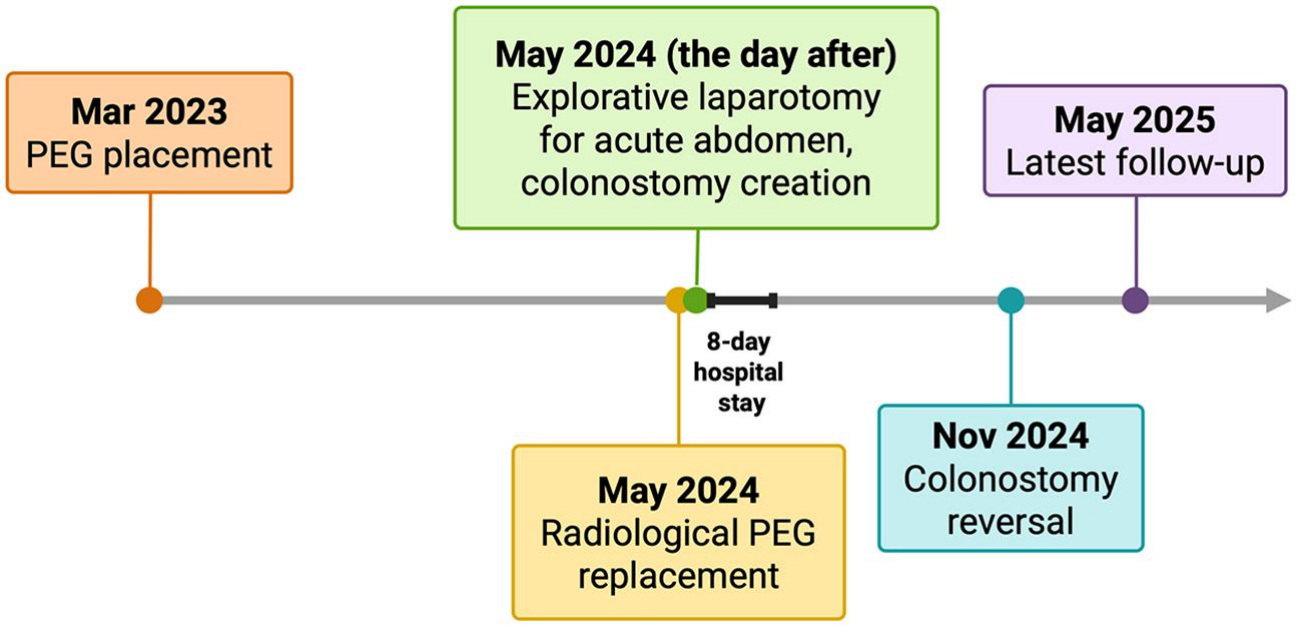
Timeline of the clinical case, reporting key events from initial presentation to the most recent outpatient follow-up. PEG, percutaneous endoscopic gastrostomy. Created in https://BioRender.com.

The clinical course after PEG placement was uneventful, with no apparent complications. He had no other significant comorbidities and no history of prior abdominal surgery. Notably, the patient had never undergone any abdominal imaging in his lifetime. In May 2024, following occlusion of the PEG catheter, a radiologic replacement was performed in an outpatient setting at the same center. The replacement procedure was entirely atraumatic: the occluded PEG was removed, and the radiologist advanced a new catheter through the existing tract, confirming correct intravisceral placement by contrast injection. The next day, the patient presented to our Acute Care Department with diffuse abdominal pain and diarrhea. On clinical examination, the abdomen was distended, with mild or poorly localized tenderness and no clear signs of peritoneal irritation. Laboratory tests showed neutrophilic leukocytosis (15.5 × 10^9^/L) and elevated C-reactive protein (135 mg/L). At admission, the differential diagnosis included bowel perforation or colonic interposition, peritonitis, disruption of the gastrocutaneous tract, intraperitoneal balloon misplacement, and gastrocolic fistula. Abdominal x-ray revealed multiple air-fluid levels and distension of both the small and large bowel. Subsequently, a contrast-enhanced computed tomography scan of the abdomen was performed, finally demonstrating the tip of the PEG catheter positioned in the mid-portion of the transverse colon (Figure 1). Leukocytosis, elevated C-reactive protein, and CT showing the PEG balloon within the transverse colon supported bowel perforation with secondary peritonitis as the most likely cause. Peritoneal tenderness was mild and poorly localized, probably blunted by Parkinson-related hyporeactivity, which delayed suspicion. Moreover, the contrast study performed during radiologic replacement may have falsely suggested proper intragastric positioning, underscoring the diagnostic pitfalls typical of neurologically compromised patients. Given the clinical and radiological findings, urgent surgical intervention was deemed necessary. An urgent laparotomy was undertaken because the patient presented with clinical and radiological signs of peritonitis. During exploration, five visceral perforations were identified: two in the sigmoid colon (entry and exit site), two in the transverse colon (entry and exit site), and one in the anterior gastric wall. The PEG tract had also caused injury to the mesosigmoid vessels, requiring segmental colectomy. A sigmoid resection with terminal colostomy was chosen instead of primary repair for both technical and physiological reasons: the local inflammation and mesenteric bleeding made simple closure unsafe, and the patient's neurologic disease was associated with a marked dolichocolon, a known predisposing factor for recurrent sigmoid volvulus (13). In addition, lateral colostomies are associated with higher rates of stomal prolapse and malfunction, particularly in patients with colonic atony or neurologic disorders (14) thus, a terminal colostomy was considered the safer option. After full recovery and stabilization of nutritional status, Hartmann's reversal was performed. Endoscopic closure techniques (TTSC, OTSC, X-Tack) and combined endo-radiologic approaches were discussed but excluded due to the multifocal, fibrotic, and contaminated nature of the lesions. Additionally, there was evidence of generalized chemical peritonitis throughout the abdominal cavity, with more pronounced acute perivisceral inflammation near the sites of perforation (Figures 2A,B). The fistulous tracts were lined with fibrotic tissue, with the involved viscera remaining partially adherent together, findings consistent with a chronic process, most plausibly arising from iatrogenic injury during the initial PEG placement. The gastric perforation was repaired by first debriding the necrotic margins and then performing a double-layer closure with absorbable suture. Colonic injuries, that were approximately 50 cm apart, were managed with a single segmental colonic resection and creation of a terminal colostomy on the transverse colon. The segment of colon between the two sites appeared macroscopically compromised, with questionable viability and signs of inflammation. For this reason, a more extensive colonic resection encompassing both perforation sites, and the intervening unhealthy segment, was deemed the safest and most appropriate approach. Extensive peritoneal lavage was performed with warm saline until the effluent was clear. Finally, a new surgical gastrostomy was performed. The colostomy became functional on postoperative day one, and enteral nutrition was started on day two without complications. The patient was discharged on postoperative day 8. He successfully underwent Hartmann's reversal six months later and is currently alive and in stable clinical condition, with a properly functioning gastrostomy. His most recent outpatient evaluation, as of May 2025, confirms his stable status. A timeline of the clinical case is presented in Figure 3.

## Discussion

Gastrostomy is commonly indicated in frail individuals burdened by significant comorbidities that compromise their ability to sustain adequate oral intake. The 30-day mortality rate following transoral PEG placement is approximately 9%–10%, but procedure-related mortality remains very low (<0.1%), as deaths are generally attributable to the patients' underlying conditions rather than the procedure itself. As reported in literature guidelines the overall complication rates are different between PEG and RIG techniques, with reported rates ranging from 0.4% to 22.5% and from 13% to 43%, respectively. The most complications are minor and manageable, including peristomal infection, as well as tube dislodgement or occlusion (7, 15, 16). The small caliber of PEG tubes increases the risk of occlusion if not flushed regularly, with reported clogging rates ranging from 20% to 45%. Although routine flushing with sterile water is generally recommended to prevent blockage, institutional practices may vary (1, 7). Notably, in our case, this mild complication fortunately prompted radiological revision one year after placement, which ultimately led to the discovery of an unrecognized underlying severe complication. A triple fistula involving the sigmoid colon, transverse colon, and stomach, has never been previously described in the literature, and its delayed clinical presentation makes the case even more exceptional. Colonic perforation is a rare, but catastrophic complication after gastrostomy. In a recent meta-analysis, it has been identified in 0.1% (310 of 272 866 patients) with PEG and 0.2% (390 of 190 851 patients) with RIG (3). Safe track needle technique, adequate endoscopic transillumination and proper finger palpation of the anterior abdominal wall are essential to minimize perforation risk during PEG placement. Additionally, in order to minimize the risk of accidental bowel interposition, full gastric insufflation is always recommended (7, 17). However, the pathophysiological dynamics of this complication remain unclear. It is plausible that the fistula was inadvertently created during the initial procedure performed the previous year. Fortunately, the fistula became walled-off and chronicized over time, thereby preventing the onset of peritonitis, while still allowing for adequate enteral nutrition. During the PEG catheter replacement, the interventional radiologist believed that the catheter had been correctly positioned. The procedure was likely stopped as soon as resistance was felt, with the assumption that the catheter tip had reached the stomach. However, in reality, the catheter had only passed through the entry and exit sites of the sigmoid colon and the entry site of the transverse colon, failing to advance further into the stomach itself. Consequently, the previously well-established fistulous tract between the transverse colon and stomach—stabilized by the catheter's presence—became disrupted and dehisced. The misplaced catheter, with the balloon inflated within the transverse colon, coupled with the subsequent resumption of enteral nutrition, caused colonic distension that led to the onset of diarrhea and abdominal pain. The latter was further exacerbated by peritonitis resulting from dehiscence of the fistulous tract and leakage of gastrointestinal contents. One possible contributing factor to the unusual anatomical configuration, with three distinct perforations involving the sigmoid colon, transverse colon and stomach, may be the presence of a dolichosigmoid in this patient. This anatomical variation, characterized by elongation and redundancy of the sigmoid colon, could have facilitated abnormal loops and adhesions over time, thereby predisposing the patient to this rare and complex pattern of injury. Additionally, Parkinson's disease is associated with significant intestinal dysmotility and delayed colonic transit, which may promote fecal stasis and progressive colonic distension. These changes may result in sigmoid colon enlargement and an increased risk of visceral interposition (18). In high-risk surgical patients, when colonic perforation is identified early, the patient remains hemodynamically stable, and there is minimal peritoneal contamination, a conservative approach may be considered. According to the American Gastroenterological Association, small, well-demarcated colonic perforations can be managed endoscopically using through-the-scope clips (TTSCs). For larger, irregular, or more complex defects, closure may be attempted with over-the-scope clips (OTSCs) or advanced suture-based systems such as the X-Tack device, provided that both appropriate expertise and specialized equipment are available (19). A case report by Al Halabi et al. describes the successful radiological replacement of a ruptured PEG catheter in two segments (20). However, that case was characterized by a technically straightforward scenario without the presence of multiple fistulas. In case of extensive tissue damage, complex fistulas, delayed diagnosis, hemodynamic instability, or signs of generalized peritonitis, surgical intervention remains the standard of care over radiologic or endoscopic approaches (19). In our case, the presence of a complex, multifocal fistula, combined with delayed diagnosis and an extensive fibrotic tissue reaction, necessitated proceeding directly with surgical management, which ultimately allowed for the safe resolution of both the colonic perforations and the PEG catheter replacement. This clinical case highlights several valuable considerations. Patients requiring PEG placement, due to their neurological comorbidities, may sometimes present with subtle or atypical clinical signs, making the assessment of abdominal symptom severity particularly challenging. Although PEG placement is generally considered a low-risk procedure, it still carries a potential risk of serious complications, such as colonic perforation.

Prevention of PEG-related colonic injury relies on adherence to simple but critical safety principles: maintaining control of the replacement tube along a mature tract, using minimal insertion force, and always confirming true intragastric position before feeding (21). Additional precautions include full gastric insufflation, clear transillumination with finger indentation, selective ultrasound in high-risk patients, and contrast verification of intragastric filling. When delayed colonic transfixion is suspected, new abdominal pain, diarrhea, or feculent output, the algorithm should be prompt: stop feeding, obtain contrast-enhanced CT, involve the surgical team early, and proceed to laparotomy if peritonitis or multiple tracts are present. Embedding this pathway within an Acute Care Surgery model ensures early recognition and coordinated management of this rare but serious complication.

This report has several limitations. It describes a single case, and no procedural imaging (endoscopic or fluoroscopic) from the initial PEG placement was available, preventing definitive confirmation of when the injury occurred. The timing of causality, whether the visceral transfixion originated during the first insertion or at replacement, therefore remains uncertain. In addition, postoperative assessment was limited to surgical outcomes; standardized measures of nutritional tolerance, aspiration risk, and quality of life were not collected. Despite these limitations, the case provides valuable insight into a rare and severe PEG-related complication and may help inform early recognition and management strategies.

We acknowledge that this case report represents an unusual scenario, however, we wish to emphasize that access to a dedicated Acute Care Department with appropriate surgical expertise is crucial for the effective management of such complex cases. In these cases, timely diagnosis and intervention are essential to optimize patient outcomes. This case serves as a reminder, and a call to action, for healthcare professionals to contribute to the evolving body of evidence surrounding PEG placement and its complications, an area of growing relevance in the context of an aging population.

## Patient's perspective

The patient expressed satisfaction with the care received, emphasizing the timeliness of the intervention and the effective management of an unexpected triple gastro-colic fistula. Although the diagnosis was initially a source of concern, clear communication, prompt investigations, and close peri-operative monitoring provided reassurance. Despite some physical discomfort and emotional distress, the patient recognized that early recognition and treatment were decisive for a favorable outcome, and expressed gratitude for the professionalism of the healthcare team.

## Data Availability

The raw data supporting the conclusions of this article will be made available by the authors, without undue reservation.
